# The default mode network and rumination in individuals at risk for depression

**DOI:** 10.1093/scan/nsad032

**Published:** 2023-05-30

**Authors:** Tina Chou, Thilo Deckersbach, Darin D Dougherty, Jill M Hooley

**Affiliations:** Department of Psychiatry, Massachusetts General Hospital, Charlestown, MA 02129, USA; Department of Psychology, Harvard University, Cambridge, MA 02138, USA; Department of Psychology, University of Applied Sciences, Diploma Hochschule, Bad Sooden-Allendorf 37242, Germany; Department of Psychiatry, Massachusetts General Hospital, Charlestown, MA 02129, USA; Department of Psychology, Harvard University, Cambridge, MA 02138, USA

**Keywords:** default mode network, rumination, neuroticism, depression, medial prefrontal cortex, inferior parietal lobule

## Abstract

The default mode network (DMN) is a network of brain regions active during rest and self-referential thinking. Individuals with major depressive disorder (MDD) show increased or decreased DMN activity relative to controls. DMN activity has been linked to a tendency to ruminate in MDD. It is unclear if individuals who are at risk for, but who have no current or past history of depression, also show differential DMN activity associated with rumination. We investigated whether females with high levels of neuroticism with no current or lifetime mood or anxiety disorders (*n* = 25) show increased DMN activation, specifically when processing negative self-referential information, compared with females with average levels of neuroticism (*n* = 28). Participants heard criticism and praise during functional magnetic resonance imaging (MRI) scans in a 3T Siemens Prisma scanner. The at-risk group showed greater activation in two DMN regions, the medial prefrontal cortex and the inferior parietal lobule (IPL), after hearing criticism, but not praise (relative to females with average levels of neuroticism). Criticism-specific activation in the IPL was significantly correlated with rumination. Individuals at risk for depression may, therefore, have an underlying neurocognitive vulnerability to use a brain network typically involved in thinking about oneself to preferentially ruminate about negative, rather than positive, information.

## Introduction

The default mode network (DMN) is a network of brain regions that is active during rest (Gusnard and Raichle, 2001; Raichle *et al.*, 2001), spontaneous cognition (e.g. Christoff *et al.*, 2009; Andrews-Hanna *et al.*, 2010a), when thinking about oneself in the past and future (e.g. Buckner and Carroll, 2007; Schacter *et al.*, 2007), and in relation to others (e.g. Saxe *et al.*, 2004; Amodio and Frith, 2006). In other words, the DMN appears to be involved in spontaneous self-referential thought. Researchers have therefore investigated this network’s activity in individuals who often have negative cognitions about themselves, such as major depressive disorder (MDD).

### Currently depressed individuals and the DMN

MDD is a mood disorder that is characterized by a persistent negative mood or loss of interest or pleasure (American Psychiatric Association, 2022). The majority of literature supports the idea that currently depressed individuals have increased resting-state functional connectivity within the DMN, whether individuals are seeking treatment for depression for the first time (Bluhm *et al.*, 2009) or experiencing depression in later life (e.g. over the age of 60 years; Alexopoulos *et al.*, 2012; Bohr *et al.*, 2012). However, there are also a few studies that show depressed individuals have decreased DMN functional connectivity at rest (e.g. Anand *et al.*, 2005; Veer *et al.*, 2010). Taken together, depressed individuals show differential engagement of this self-referential network when they are not being asked to do a specific task in the MRI scanner other than letting their minds freely wander, relative to non-depressed individuals.

 Depressed individuals not only show differential DMN connectivity at rest but also show differential DMN activation when asked to do a task. Individuals with depression have shown widespread increased activation in DMN regions such as the hippocampus, ventromedial prefrontal cortex, anterior cingulate cortex (ACC) and lateral parietal cortex (LPC) when viewing negative pictures relative to controls, but there were no differences in activation in the posterior cingulate cortex (PCC) or precuneus (Sheline *et al.*, 2009). Conversely, depressed individuals have also shown widespread decreased activation in the dorsomedial prefrontal cortex, supragenual ACC and precuneus compared to controls when making judgments about self-relatedness of negative pictures (Grimm *et al.*, 2009). This pattern of findings suggests that DMN activity is specifically linked with the preferential processing of negative information in individuals who currently experience clinical levels of depressive symptoms.

### Individuals at risk for depression and the DMN

There is some evidence that individuals who are at risk for depression also have differential DMN activity. Researchers have found a negative correlation between the left amygdala volume and the medial prefrontal cortex (MPFC) thickness in community sample participants who scored highly on a composite measure of negative affect (which included measures of neuroticism, anxiety, behavioral inhibition, mood disturbance and harm avoidance; Holmes *et al.*, 2012). They also found that individuals with high polygenic risk for depression have a reduced left MPFC cortical thickness. This study provides some evidence that individuals at risk for depression show structural differences relative to controls in at least one DMN region.

Another risk factor for depression is a family history of depression, which has been linked to greater resting-state DMN connectivity within the left LPC and precuneus/PCC (Posner *et al.*, 2016). However, in Posner *et al.* (2016), many of these individuals also had current or past history of depression, anxiety and/or psychotropic medication usage. Therefore, their results may reflect DMN connectivity patterns associated with current clinical levels of depression or recovery from depression. To answer the question of whether differential DMN activity reflects an underlying cognitive vulnerability for depression, it is important to study DMN activity in individuals at risk for depression, but who have no current or lifetime history of depression or psychotropic medication usage.

### Rumination in depression and the DMN

Researchers have primarily focused on rumination, negative and passive self-focused thoughts, as the specific type of cognition that is possibly associated with DMN abnormalities. Rumination can predict the onset of depressive episodes and depressive symptoms (Nolen-Hoeksema, 2000). The literature is mixed in terms of the specific kind of rumination and correlations with different DMN regions. Connectivity between a DMN region, the PCC and the subgenual cingulate has been positively correlated with the tendency to ruminate and ruminative brooding in depressed individuals and healthy controls (Berman *et al.*, 2011). Depressive rumination in depressed individuals has been positively correlated with connectivity between the lateral temporal cortex (LTC) and the parahippocampal cortex (Zhu *et al.*, 2017). Hamilton *et al.* (2011) found that DMN activity in depressed individuals, relative to activity in the task-positive network (a network that demonstrates increased activity during tasks that require attention and is anticorrelated with the DMN; Fox *et al.*, 2005), was positively correlated with depressive rumination and negatively correlated with reflective rumination. Finally, functional connectivity between the MPFC and the ACC has been positively correlated with rumination (Zhu *et al.*, 2012).

### Present study

Differential patterns of DMN activation and connectivity at rest and during tasks with negative stimuli appear early in MDD and may persist as people continue to suffer from depression. In depressed individuals, DMN activity appears to be involved in rumination. In the present study, we investigated whether at-risk individuals also show differential DMN activation and if this is also associated with rumination in individuals who are at risk for, but who have not yet developed, depression. Our at-risk sample consisted of females with high levels of neuroticism. Although being female and having high neuroticism are risk factors for depression and anxiety (Clark and Watson, 1991; Vink *et al.*, 2008; Faravelli *et al.*, 2013), numerous studies have shown that women are at higher risk for developing depression compared to males (Galambos *et al.*, 2004; Piccinelli and Wilkinson, 2018). Several large longitudinal studies have shown that high levels of neuroticism are significant predictors of the first onset of a depressive episode, even after controlling for sex (e.g. Kendler *et al.*, 2006, 1993a,b). A meta-analysis of 10 prospective studies including *n* = 117 899 participants showed that high neuroticism was associated with increased risk for depressive symptoms and current depressive symptom severity and current depression also significantly increased levels of neuroticism (Hakulinen *et al.*, 2015).

## Methods

### Participants

**Fig. 1.**
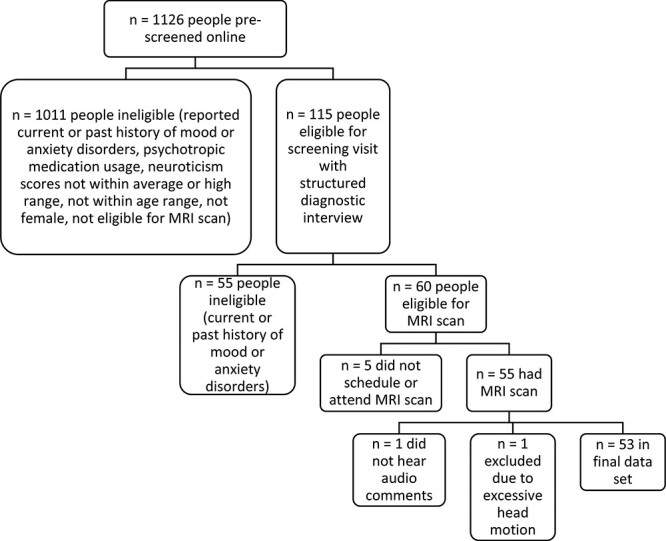
Flow chart showing the screening process for the final sample.

All participants provided written informed consent in accordance with the Declaration of Helsinki, and the study was approved by Harvard University’s Institutional Review Board. 1126 individuals were screened for this study (Figure 1). Only females who scored in the upper 80th percentile (high neuroticism or at-risk group) or in the 40th to 60th percentile (average neuroticism or control group) on the neuroticism items from the NEO-Five Factor Inventory (FFI) (Costa and McCrae, 1992) were brought in to the laboratory for further screening (*n* = 115 individuals). These individuals were screened for current psychotropic medication usage and current and lifetime history of mood and anxiety disorders using the Structured Clinical Interview for the Diagnostic and Statistical Manual of Mental Disorders (DSM-5), Research Version (First *et al.*, 2015). Based on this interview, *n* = 60 individuals had no current or lifetime history of mood or anxiety disorders. Five participants did not want to schedule or did not show up for the MRI scan. Therefore, *n* = 55 participants were scanned. One participant was excluded because she did not hear any of the auditory stimuli due to technical issues. Another participant was excluded from analyses because of excessive head motion. The final dataset therefore consisted of *n* = 28 females with average levels of neuroticism (control group) and *n* = 25 females with high levels of neuroticism (at-risk group). The control and at-risk groups did not differ in age [at-risk *M *= 20.36 years, Standard Deviation (SD) = 2.22 years, controls *M* = 21.00 years, SD = 2.37 years; *t*(51) = 1.01, *P* = 0.32] or years of education [at-risk *M* = 14.32 years, SD = 2.10, controls *M* = 14.49 years, SD = 1.75 years; *t*(51) = 0.51, *P* = 0.61]. Participants self-identified as African American (11.9%), Asian (35.6%), Caucasian (35.6%), Hispanic (5.1%) and multiracial (11.9%).

### Measures

#### NEO-FFI

To assess neuroticism, we administered the NEO-FFI (Costa and McCrae, 1992), a 60-item self-report measure of the Big Five personality traits: extraversion, agreeableness, conscientiousness, neuroticism and openness to experience. Example neuroticism items include ‘Sometimes I feel completely worthless’, ‘I am seldom sad or depressed’ and ‘I often feel tense and jittery’. This measure had good levels of internal consistency (α = 0.84) in our sample.

#### Ruminative responses scale

The Ruminative Responses Scale (RRS) of the Response Styles Questionnaire (Nolen-Hoeksema and Morrow, 1991) is a 22-item self-report measure that assesses the frequency of ruminative thinking. Participants are asked to rate on a 1 to 4 scale, where 1 = almost never and 4 = almost always, how often they endorse the statement when they are feeling sad. The total score ranges from 22 to 88, with higher scores indicating higher levels of rumination. This measure had high levels of internal consistency (α = 0.95) in our sample.

### MRI data acquisition

Participants were scanned using a 3.0 Tesla Siemens MAGNETOM Prisma MRI scanner (Siemens, Erlangen, Germany) at the Center for Brain Science Neuroimaging Facility at Harvard University. The scanner underwent a VE11C software upgrade after 28 subjects had been scanned; software version and scanner were entered as regressors. Functional blood-oxygen-level-dependent MRI images were acquired using a gradient echo T2*-weighted sequence (reptition time (TR)/echo time (TE)/flip angle = 650 m/34.4 ms/54°) with an in-plane resolution of 2.3 × 2.3 × 2.3 mm. T1-Weighted high-resolution images (TR/TE/flip angle = 2200 ms/1.57 ms/7°) with an in-plane resolution of 1.2 × 1.2 × 1.2 mm were collected and used for co-registration with fMRI data.

### Critical comments

Participants heard a stimulus set of comments that were developed in Dr Jill Hooley’s laboratory adapted from comments made by mothers of individuals with depression and borderline personality disorder (Hooley *et al.*, 2005, 2010, 2012). These comments have been used in numerous studies evaluating the effects of criticism (De Raedt *et al.*, 2017; Baeken *et al.*, 2018; Nook *et al.*, 2018; Dedoncker *et al.*, 2019). The comments were 30 s in duration and involved blocks of four critical and praise comments. Critical comments included phrases such as ‘one of the things that bothers me about you is that you’re not very considerate of other people. You can be very self-involved at times. […] It’s all about you and what you need’. Praise comments included phrases such as ‘one of the things that I really like about you is your sense of humor. It’s not that you’re always telling jokes or anything like that. But you can be really really funny’. To increase the potential emotional impact of hearing the comments, participants were instructed ‘we would like you to imagine that the following comments are being said to you by someone who is very important in your life’. The order of the critical or praise comment blocks was also counterbalanced across participants.

### Data analyses

Functional data were processed using SPM8 software (Wellcome Department of Cognitive Neurology, London, UK). For each individual subject, fMRI images were realigned to a reference image (the image in the middle of the time series) using a six-parameter rigid body spatial transformation and a least squares approach, co-registered to their high-resolution structural scan, spatially/stereotactically normalized to the standardized normalized space established by the Montreal Neurological Institute (MNI; http://www.bic.mni.mcgill.ca) and then smoothed/convolved with a three-dimensional Gaussian filter of 6-mm full-width at half maximum (FWHM). This was done to reduce noise due to residual differences in anatomy during group averaging. It should be noted that the default FWHM of the Gaussian smoothing kernel is 8 mm. Therefore, our Gaussian filter of 6 mm was more conservative. Participants in the at-risk and control groups did not significantly differ in motion estimates such as maximum absolute motion [at-risk *M* = 0.77, SD = 0.45; controls *M* = 0.81, SD = 0.52; *t*(51) = 0.05, *P* = 0.83]. Note that maximum absolute motion values less than 1.49 are desirable. The at-risk group and controls had fewer than 5 movements > 0.5 mm (which indicates good data; the one at-risk participant who had greater than five movements was excluded from the final dataset as previously mentioned in the Participants section).

Individuals’ preprocessed images were then entered into first level, within-subject analyses. During the realignment preprocessing step, a set of realignment parameters representing movement (*x*, *y* and *z* denote pitch, roll and yaw) were generated and we included these parameters as covariates to further correct for motion during the individual first-level analyses. Contrast images for conditions of interest (rest periods before criticism, rest periods after criticism, rest periods before praise and rest periods after praise) were created for each individual subject. The rest periods, rather than during the comments themselves, were the conditions of interest because first, the majority of studies identifying differential DMN activity have looked at DMN activity during rest, and we wanted to understand if at-risk individuals showed differential activation during rest periods after criticism and praise. Second, we were most interested in activation associated with participants actually processing the comments, which was more likely to occur during the open periods of rest after the comments when participants were not being asked to do anything in particular (as opposed to during the comments where they were instructed to imagine that the comments were being spoken by an important person in their life). The contrast images for each individual subject were then entered in a second level, between-group, random effects model. We created a general linear model flexible factorial model with two factors [group (at-risk individuals and controls) and condition (rest periods after criticism, rest periods before criticism, rest periods after praise and rest periods before praise)]. We then computed contrast images by creating contrast vectors for our contrasts of interest [(i) at-risk individuals > controls rest periods after criticism > rest periods before criticism; (ii) at-risk individuals > controls rest periods after praise > rest periods before praise]. Significant clusters of activation for each of these contrasts were associated with a *Z*-score test statistic.

We completed region of interest (ROI) analyses at *P* < 0.005 with our a priori regions as defined by anatomical masks from the Wake Forest University Pick Atlas (Maldjian *et al.*, 2003). Our a priori ROIs were based on identification of Buckner *et al.* (2008) of core regions in the DMN: the MPFC (Brodmann areas 9, 10, 24 and 32), PCC/retrosplenial cortex (Brodmann areas 29, 30/23 and 31), hippocampal formation (HF; bilateral hippocampus and HF), LTC (Brodmann area 21) and inferior parietal lobule (IPL; Brodmann areas 39 and 40). We used Analysis of Functional NeuroImages (AFNI’s) 3DClustSim (Forman *et al.*, 1995; Cox, 1996) to control for multiple statistical comparisons. This program estimates the probability of a false detection of a significant activation of a certain cluster size (i.e. what is the probability that a specific cluster size is truly significant). More specifically, when clusters of activation were identified as being significant at *P* < 0.005, 3DClustSim was then used to determine if the size of the cluster survived a cluster-level positive detection rate at *P* < 0.05. We have previously used this method to correct for multiple comparisons (Dougherty *et al.*, 2016; Chou *et al.*, 2022).

### Cognitive measures analyses

Spherical ROIs (radius = 5 mm) were created around peak voxels of activation in DMN regions, which showed increased criticism-specific activation in the at-risk individuals, the MPFC and the IPL. MarsBaR, a ROI toolbox for SPM (http://marsbar.sourceforge.net; Brett *et al.*, 2002), was used to extract beta weight values from these ROIs for each subject for each condition of interest. Due to the potential effect of outliers on correlation coefficients, two controls who scored greater than 2 s.d. above the control group’s mean RRS rumination score (>58) were removed from correlational analyses. We then conducted Pearson’s *r* correlational analyses in SPSS Version 24 with the MPFC and IPL beta weight values to investigate the relationship between activation in the MPFC and the IPL and rumination.

## Results

### DMN hyperactivation specific to criticism, not praise

**Fig. 2.**
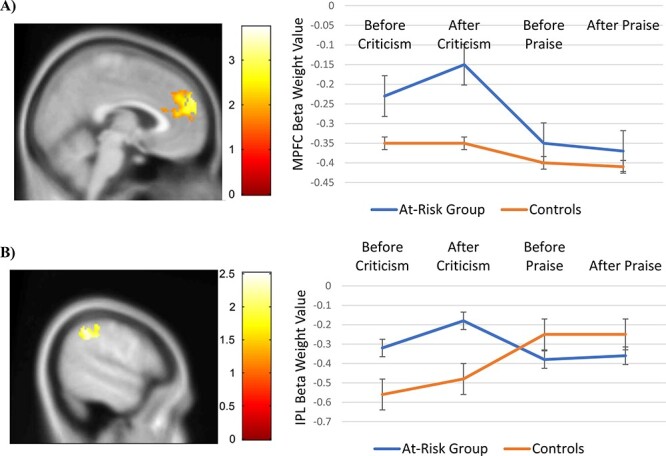
Individuals at risk for depression showed significantly greater activation in two DMN regions: (A) the MPFC and (B) the IPL, after hearing criticism (3DClustSim corrected *P* < 0.05), relative to controls. There were no significant group differences in DMN activation after hearing praise.

After hearing the critical comments, individuals at risk for depression (i.e. females with high levels of neuroticism) showed greater activation in two DMN regions, relative to controls (Figure 2). Specifically, individuals at risk for depression showed greater activation in bilateral MPFC [left MPFC (Brodmann area 10), MNI coordinates = −6, 60, 18, *Z*-score = 3.69, *k* = 81 voxels, right MPFC (Brodmann area 9), MNI coordinates = 6, 58, 30, *Z*-score = 3.35, *k* = 61 voxels] after hearing criticism. These clusters of activation exceeded 3DClustSim correction for multiple comparisons to preserve an α < 0.05. At-risk individuals were not significantly different from controls in MPFC activation after hearing praise (Figure 2).

At-risk individuals showed increased activation in the left IPL (Brodmann area 40; MNI coordinates = −46, −36, 50, *Z*-score = 2.47, *k* = 212 voxels) after hearing criticism, compared to controls. This cluster of activation exceeded 3DClustSim correction for multiple comparisons to preserve an α < 0.05. There were no significant differences in IPL activation between the at-risk individuals and controls after hearing praise (Figure 2).

### DMN hyperactivation and rumination

**Fig. 3.**
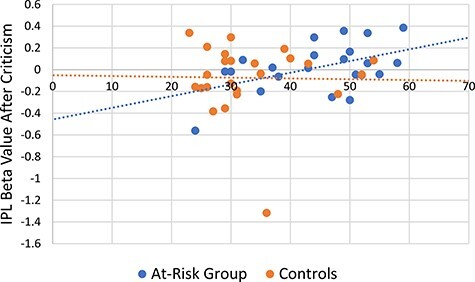
IPL activation after criticism was significantly correlated with rumination in individuals at risk for depression [Pearson’s *r*(25) = 0.479, *P* = 0.02]. Rumination was not correlated with criticism-specific IPL activation in controls [Pearson’s *r*(26) = −0.020, *P* = 0.93].

Activation in the left IPL after criticism was correlated with rumination in the at-risk individuals [Pearson’s *r*(25) = 0.479, *P* = 0.02; Figure 3], which was not the case for controls [Pearson’s *r*(26) = −0.020, *P* = 0.93]. Criticism-specific activation in the left MPFC was not correlated with rumination [at risk: Pearson’s *r*(25) = 0.245, *P* = 0.27; controls: Pearson’s *r*(25) = 0.291, *P* = 0.17]. Activation in the right MPFC after criticism was also not correlated with rumination [at risk: Pearson’s *r*(25) = 0.121, *P* = 0.59; controls: Pearson’s *r*(25) = 0.337, *P* = 0.11].

## Discussion

Currently depressed individuals have shown increased and decreased resting-state DMN functional connectivity relative to controls. In this study, individuals identified as being at risk for depression (females with high levels of neuroticism) show some evidence of increased DMN activation after hearing criticism, relative to individuals with average levels of neuroticism. Specifically, at-risk individuals demonstrated greater activation in bilateral MPFC and the left IPL (Figure 2). As with previous studies, we found evidence of differential activation in specific DMN regions (MPFC and IPL) rather than across the entire DMN. Our MPFC hyperactivation finding is in line with the results from Holmes *et al.* (2012), which linked the reduced MPFC cortical thickness to individuals at risk for depression. Posner *et al.* (2016) implicated greater connectivity within the left LPC and precuneus/PCC in individuals at risk for depression; our at-risk individuals did not show hyperactivation in either of these regions. However, they included individuals with current and lifetime psychological disorders. Based on this pattern of findings, it is possible that differences in MPFC structure and activity could be an underlying vulnerability for individuals at risk for depression, but who have never experienced depression before.

We found greater activation specifically within the dorsal MPFC (DMPFC), not ventral MPFC (VMPFC), after criticism in the at-risk individuals, which extends previous work suggesting that the DMPFC, in particular, is specifically involved when thinking about positive or negative information in relation to oneself (Andrews-Hanna *et al.*, 2010b). Based on this, researchers have suggested that the DMN can be divided into two distinct subsystems, a medial temporal lobe subsystem (which includes the VMPFC) and a DMPFC subsystem, with the DMPFC subsystem supporting self-referential thinking and the medial temporal lobe subsystem being involved in memory-based decisions about the future (Andrews-Hanna *et al.*, 2010b). Previous studies have shown that the DMPFC appears to be involved in comparing incoming self-referential information to personal standards, whereas the VMPFC is involved in switching attention to self-relevant information (Lemogne *et al.*, 2012). The fact that the at-risk individuals showed greater DMPFC, and not VMPFC activation, specifically after criticism and not praise, suggests that the individuals at risk for depression may have especially been considering the self-relevance of the negative information, rather than positive information.

Individuals at risk for depression also had increased IPL activation after criticism. The IPL, as part of a larger ‘semantic network’ that largely overlaps with the DMN, has been associated with semantic processing, the ability to store and fluidly manipulate knowledge about the world (e.g. Binder *et al.*, 2009). The IPL has also been linked to conceptual integration, as demonstrated by the finding that the IPL only shows activation at the end of sentences and after comprehension of a sentence as a whole (Humphries *et al.*, 2006). Binder *et al.* (2009) point out that this region is relatively nonexistent in lower primates and is primarily anatomically connected to other association regions of the brain, not primary sensory areas. Taken together, the fact that at-risk individuals showed increased IPL activation after hearing criticism may mean that these individuals were more inclined to engage in higher-order cognitive processing of the critical comments relative to controls.

At-risk individuals did not show any differences in activation in the HF or PCC/retrosplenial cortex after criticism, relative to controls. This is in contrast to a previous finding that depressed adolescents show increased activation in the parahippocampal gyrus after hearing criticism from their own mothers (Silk *et al.*, 2017). Another study in healthy adolescents showed increased activation in the parahippocampal gyrus after hearing neutral comments as opposed to criticism from their mothers, as well as decreased activation in the PCC after maternal criticism (Lee *et al.*, 2014). On the other hand, our finding is consistent with a study with depressed individuals showing no differences in activation in the PCC when viewing negative pictures relative to controls (Sheline *et al.*, 2009). It is possible that our null findings are due to differences in types of negative study stimuli. The hippocampus is involved in memory processing (e.g. Eichenbaum, 2001), and the PCC has been implicated in episodic memory retrieval and autobiographical memories of family members and friends (Maddock *et al.*, 2001). Hearing comments from their own mothers may have prompted participants in the studies by Silk *et al.* (2017) and Lee *et al.* (2014) to think about past instances during which they may have acted in ways that reflected the behaviors in the comments. They may also have had other memories associated with their mothers criticizing them or recalled other specific memories involving their mothers. We asked participants in the present study to imagine that both the critical and praise comments were being spoken by someone close to them; however, participants may have had varying levels of success in doing so. Although both groups separately showed HF activation during the rest periods after criticism, it is possible that participants in the present study may not have engaged in additional retrieval of memories associated with the person saying the comments, which could have led to a group difference in HF (and PCC) activation after hearing criticism. A future study similarly using an at-risk sample exposed to critical and praise comments from their own mothers would help disentangle whether these differences are due to differences in type of stimuli.

We also did not find significant group differences in LTC activation after criticism or praise. It is possible that differential LTC activation associated with emotional stimuli is specific to current depression, rather than in individuals who are at risk for, but who have never experienced, depression; previous studies in depressed individuals have shown that they do not deactivate the LTC when passively viewing and reappraising negative pictures (Sheline *et al.*, 2009) and have decreased LTC activation during positive stimuli (Fitzgerald *et al.*, 2008). Another study has found that LTC connectivity was correlated with depressive rumination in depressed individuals (Zhu *et al.*, 2017). Longitudinal studies with participants before and after the first onset of a depressive episode could specifically investigate whether there are changes in LTC activation with depressive episode status.

We have shown that individuals at risk for depression do not necessarily show differential DMN activation during all periods of rest. Specifically, these individuals demonstrate greater MPFC and IPL activation relative to controls during rest periods after hearing criticism; they did not differ from controls in their DMN activation during rest periods after hearing praise. Our results are in line with a study by Sheline *et al.* (2009) with depressed individuals showing increased DMN activation when viewing negative pictures relative to controls. We have thus extended these findings by showing greater DMN activation after negative self-referential stimuli (i.e. criticism) in at-risk individuals.

In terms of the specific cognition associated with DMN activation, criticism-specific IPL activation was significantly correlated with rumination. We have extended previous findings showing significant correlations between DMN functional connectivity and rumination; in this study, we have found a significant correlation of medium to large effect size (Pearson’s *r* > 0.30) between rumination and DMN activation. We have previously found that transcranial direct current stimulation of the IPL can reduce negative thoughts about the past in a community sample (Chou *et al.*, 2020). Future studies may therefore investigate using neuromodulation of the IPL as an intervention for rumination in at-risk individuals.

There were several limitations to this study. First, it should be acknowledged that our controls, although scoring in the average range on one risk factor for depression, neuroticism, could have other risk factors for depression that were not assessed. For example, we did not assess family history of depression in our controls. If our controls had a family history of depression, then they would more accurately be categorized as at-risk individuals, not controls. It would therefore be difficult to meaningfully interpret our group difference findings. At the same time, Holmes *et al.* (2012) found that controlling for family history of psychiatric disorders did not change the reduced MPFC cortical thickness findings in high negative affect individuals. Future studies involving a more extensive screening of multiple risk factors for depression are necessary to better understand the relationship between DMN activity and specific *vs* general risk for depression.

Second, neuroticism and being female are non-specific risk factors for depression; they are also risk factors for anxiety disorders (Clark and Watson, 1991); therefore, all our results could be considered as possibly applying to individuals at risk for anxiety disorders. It should be noted that since depression and anxiety are highly comorbid (Kaufman and Charney, 2000; Brown *et al.*, 2001; Lamers *et al.*, 2011), it is not unusual that they have shared risk factors. Individuals with anxiety disorders have also been found to have differential DMN activity relative to controls (Zhao *et al.*, 2007; Gentili *et al.*, 2009; Sylvester *et al.*, 2012). Future studies could recruit based on a risk factor that is specific to depression and not other disorders such as anxiety.

Third, we assessed the relationship between rumination and DMN activation by using a self-report measure of the tendency to ruminate; it is possible that participants were not ruminating during the periods of DMN hyperactivation. Future studies with thought sampling in the moment would help clarify the specific forms of negative cognition supported by DMN activity. Finally, our final sample size was relatively small; future investigations should attempt to replicate our findings in a larger sample.

## Conclusions

Overall, our results suggest that individuals at risk for depression may use a self-referential brain network when preferentially processing negative, rather than positive, information. This form of biased processing is associated with ruminative thoughts and may reflect an underlying neurocognitive vulnerability for later depression. Future treatments targeting the MPFC or the IPL could serve as a preventative intervention for individuals at risk for depression.
